# Threshold-Voltage Extraction Methods for Atomically Deposited Disordered ZnO Thin-Film Transistors

**DOI:** 10.3390/ma16082940

**Published:** 2023-04-07

**Authors:** Minho Yoon

**Affiliations:** Department of Physics and Institute of Quantum Convergence Technology, Kangwon National University, Chuncheon 24341, Republic of Korea; minhoyoon78@gmail.com

**Keywords:** threshold voltage, localized trap states, ZnO, thin-film transistors

## Abstract

In this paper, we present a threshold-voltage extraction method for zinc oxide (ZnO) thin-film transistors (TFTs). Bottom-gate atomic-layer-deposited ZnO TFTs exhibit typical n-type enhancement-mode transfer characteristics but a gate-voltage-dependent, unreliable threshold voltage. We posit that this obscure threshold voltage is attributed to the localized trap states of ZnO TFTs, of which the field-effect mobility can be expressed as a gate-bias-dependent power law. Hence, we derived the current–voltage relationship by dividing the drain current with the transconductance to rule out the gate-bias-dependent factors and successfully extract the reliable threshold voltage. Furthermore, we investigated the temperature-dependent characteristics of the ZnO TFTs to validate that the observed threshold voltage was genuine. Notably, the required activation energies from the low-temperature measurements displayed an abrupt decrease at the threshold voltage, which was attributed to the conduction route change from diffusion to drift. Thus, we conclude that the reliable threshold voltage of accumulation-mode ZnO TFTs can be determined using a gate-bias-dependent factor-removed current–voltage relationship with a low-temperature analysis.

## 1. Introduction

Oxide semiconductors such as zinc oxide (ZnO), indium–gallium–zinc oxide (IGZO), and indium–zinc oxide (IZO) have been extensively investigated for their application in transparent electronics, the backplanes of large-area active-organic light-emitting-diode displays, and liquid crystal displays due to their high mobility, high transparency to visible light, and low processing temperatures [1,2,3,4]. However, oxide-semiconductor-based thin-film transistors (TFTs) suffer from severe threshold shifts during operation; therefore, compensation circuits are generally incorporated in their applications [5,6]. The threshold voltage (*V_th_*) of TFTs is a gate voltage required to turn the devices on and is estimated using several methods, including a commonly used linear fitting method [7,8], a field-effect mobility derivation method [9], and a constant-current method [10]. However, the definition of the threshold voltage of TFTs is ambiguous. In the case of metal–oxide–semiconductor field-effect transistors (MOSFETs), the threshold voltage is defined as a gate voltage for the strong inversion point [11]. However, TFTs usually operate in an accumulation mode; hence, the threshold voltage of TFTs cannot be defined as in the case of MOSFETs. In some sense, the flatband voltage, a gate voltage for the zero-semiconductor band bending point, could be a more appropriate terminology for TFTs [12]. However, the threshold voltage of TFTs is used in the same way as that of MOSFETs. In addition, the near-threshold voltage operation of TFTs has received considerable attention for low-power applications [13]. Thus, the in-depth understanding of charge-transport behaviors’ near-threshold voltage and the definition of the threshold voltage of TFTs are quite important. Indeed, due to localized states in the disordered oxide semiconductors, the charge-transport characteristics of TFTs are severely affected by interface and semiconductor trap states [14,15]. As a result, it is difficult to determine the conduction states and threshold voltage. Efforts to resolve these issues have led to several proposed approaches, such as the charge- and surface-potential-based compact models [16,17,18] and the semi-empirical model based on multi-frequent capacitance–voltage measurements [19]. These approaches generally yield promising results, but it seems that more reliable and practical methods are required to determine the threshold voltage for the disordered oxide semiconductor-based TFTs.

In this study, we explored the threshold-voltage extraction method and charge-transport behaviors near the threshold voltage of atomic-layer-deposited ZnO TFTs. Bottom-gate, atomic-layer-deposited ZnO TFTs show typical n-type enhancement-mode transfer characteristics with a field-effect mobility of 3.1 cm^2^V^−1^s^−1^. However, defining the threshold voltage with the linear fitting method seems unreliable; as the gate bias increases, the threshold voltage changes. We posit that the localized states of ZnO TFTs are attributed to this ambiguous threshold voltage, of which the field-effect mobility is modeled as a power law [20,21,22]. In order to extract the reliable threshold voltage, we modified the current–voltage relationship by dividing the drain current with the transconductance. Following the modification, the gate-voltage-independent and reliable threshold voltage was successfully extracted. In addition, in order to ensure that the threshold voltage was genuine, we investigated the activation energies as a function of the gate voltage from the temperature-dependent transfer characteristics of the ZnO TFTs. The activation energy significantly decreased at the threshold voltage, which was attributed to the conduction route change from diffusion to drift. Therefore, we believe that the reliable threshold voltage of disordered ZnO TFTs can be determined by the proposed method.

## 2. Materials and Methods

**Figure 1:**
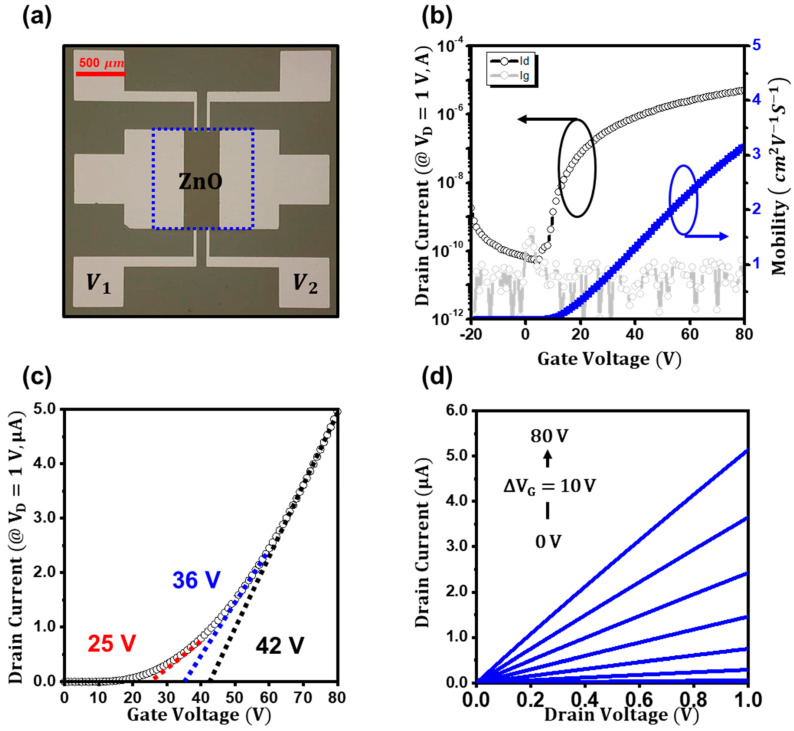
(**a**) Optical microscopy image of the ZnO TFTs with a four-point probe configuration. (**b**) Transfer characteristics (*I_ds_* vs. *V_gs_*) of the ZnO TFTs. (**c**) The threshold-voltage extraction of the ZnO TFTs with the gate bias range up to 40 (red), 60 (blue), and 80 (black) V, respectively. (**d**) Output characteristics (*I_ds_* vs. *V_ds_*) of the ZnO TFTs.

Bottom-gate top-contact (BGTC) ZnO TFTs were fabricated as shown in the optical microscope image in Figure 1a. A 200 nm thick p^+^-Si/SiO_2_ wafer coated with a 10 nm thick Al_2_O_3_ was used as the substrate, and a 6 nm thick ZnO film was deposited by cyclic atomic layer deposition (ALD) at 80 °C using diethylzinc (DEZ, Aldrich, St. Louis, MO, USA) and water as zinc and oxygen precursors, respectively. After patterning the ZnO layer by a conventional lift-off process, 50 nm thick Al source and drain electrodes were deposited by thermal evaporation and patterned using a shadow mask, which had a width and length of 1000 and 360 μm, respectively. To extract the intrinsic conductance of the ZnO channel, a four-point probe source and drain configuration was used, of which the voltage-probing electrodes (*V*_1_ and *V*_2_) were placed at 120 and 240 μm in the channel. The geometric capacitance of the dielectric was measured to be 16.4 nF cm^−2^ at 1 kHz, using an LCR meter (HP4284A, Agilent Technologies, Santa Clara, CA, USA). The thickness of the films was measured with an ellipsometer (AutoEL-II, Rudolph Research, Hackettstown, NJ, USA) and confirmed with an atomic force microscope (XE100, Park Systems, Suwon, Korea). A magnetic-sector-based instrument (TOF.SIMS-5, ION-TOF) was used for time-of-flight secondary ion mass (SIMS) analysis, with a Bi^+^-ion beam (30 keV) as the primary ion source to detect negative secondary ions. For the depth profiling, the etching source of Cs^+^ (1 keV) was used and the analysis area was 100 × 100 μm^2^. The homemade Hall measurement system was used for analyzing the ZnO films; the electromagnet (up to 1 T) was placed beneath the probing stage, and the electrical signals were recorded with a semiconductor parameter analyzer (Model HP4155C, Agilent Technologies). The current–voltage (*I–V*) characteristics of the transistors were also investigated with the semiconductor parameter analyzer (Model HP4155C, Agilent Technologies). A liquid-nitrogen cooling cryostat was used for temperature-variable current–voltage measurements. The temperature range was from 180 to 300 K. The extrinsic field-effect mobility of the TFTs in a linear regime was estimated by the following equation:

(1) $$\mu_{lin}=\frac{1}{C_{i}V_{ds}}\frac{L}{W}\frac{\partial I_{ds}}{\partial V_{gs}}$$

where *I_ds_* is the drain current, *V_gs_* is the gate voltage, *C_i_* is the geometric dielectric capacitance, *V_ds_* is the drain voltage, and *L* and *W* are the channel length and width, respectively.

## 3. Results

Figure 1b displays the typical n-type transfer characteristics (*I_ds_* vs. *V_gs_* at *V_ds_* = 1 V) of the ZnO TFTs [23]. As the gate bias increased, the electron current (*I_ds_*) increased from ~10^−11^ to ~10^−6^ A, and the field-effect mobility from the transconductance also gradually increased, reaching a maximum of 3.1 cm^2^V^−1^s^−1^ at *V_gs_* = 80 V. However, the threshold voltage (*V_th_*) of ZnO TFTs was difficult to determine. As shown in Figure 1c, using the widely used linear fitting method, the threshold voltage can be estimated to be 25 V when extracted up to the gate bias of 40 V, but it increased to 36 and 42 V when extracted up to the gate bias of 60 and 80 V, respectively. The threshold voltage of TFTs is a widely used key parameter for investigating the interface trap states and the stability of the devices [24,25]. Thus, if the threshold voltage is not reliable, as in ZnO TFTs, the analysis of the devices leads to misinterpreted consequences. The current–voltage characteristics of ZnO TFTs with a two-terminal geometry are the sum of the channel resistance (*R_ch_*) and the contact resistance (*R_c_*). If the contact resistance (*R_c_*) is not negligible, the threshold voltage may be ambiguous, as in ZnO TFTs, because the contact resistance decreases with the increasing gate voltage [26,27]. To address this issue, as schematically illustrated in Figure S1a, the contact-effect-removed current–voltage characteristics of the ZnO TFTs were investigated by measuring the potential distribution in the channel; then, the intrinsic threshold voltage was revisited. However, the obscure threshold voltage can still be seen in Figure S1b, which was 25, 37, and 46 V for the gate biases of 40, 60, and 80 V, respectively. Figure 1d shows the corresponding output characteristics (*I_ds_* vs. *V_ds_*) of the ZnO TFTs, which revealed an ohmic contact behavior.

As reported elsewhere, charges in amorphous or polycrystalline disordered semiconductors move through localized states via thermally activated hopping [28,29,30]. Thus, as the gate bias increases, the localized states are occupied by the induced charges, resulting in increased field-effect mobility, modeled as the power law *μ = μ*_0_ *(V_gs_* − *V_th_)^α^*, where α is the power law exponent. As a result, the drain current (*I_ds_*) can be expressed as the gate-bias-dependent power law *I_ds_* = *μ*_0_ *C_i_ WL*^−1^*(V_gs_* − *V_th_)^α^*^+1^*V_ds_*. Thus, the widely used linear fitting method results in the arbitrary extraction of ZnO TFTs because the drain current (*I_ds_*) is not linearly dependent on the gate bias. Hence, we attempted to modify the threshold-voltage extraction method based on the power-law-dependent relations. By expressing the drain current (*I_ds_*) and transconductance (*g_m_*) of the TFTs as gate-bias-dependent power law equations, a power-law term-removed current relation can be derived by dividing the drain current with the transconductance, as in the following equation. Using this relation, the exponent of α and the threshold voltage can be estimated.

(2) $$I_{ds}=\mu_{0}C_{i}\frac{W}{L}{\left(V_{gs}-V_{th}\right)}^{\alpha +1}V_{d}$$

(3) $$g_{m}=\frac{\partial I_{ds}}{\partial V_{g}}=\left(\alpha +1\right)\mu_{0}C_{i}\frac{W}{L}{\left(V_{gs}-V_{th}\right)}^{\alpha }V_{d}$$

(4) $$\frac{I_{ds}}{g_{m}}=\frac{\left(V_{gs}-V_{th}\right)}{\left(\alpha +1\right)}$$

**Figure 2:**
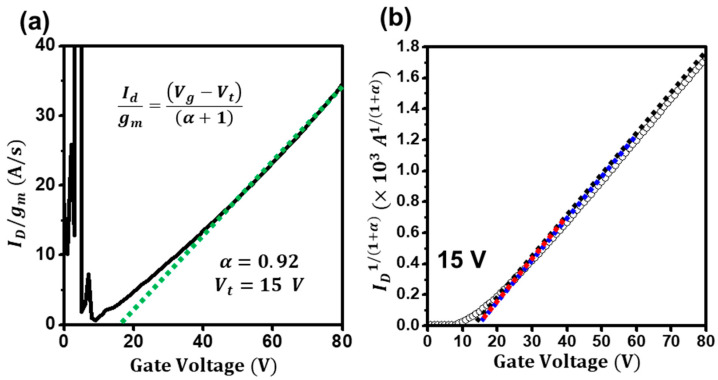
(**a**) The modified plot of *I_d_/g_m_* for the ZnO TFTs. (**b**) The power-law-term-removed threshold-voltage extraction of the ZnO TFTs.

Figure 2a shows the extraction plot of *I_ds_/g_m_* for the ZnO-based TFTs. From the linear fitting of the plot, the exponent of α and the threshold voltage were estimated to be 0.92 and 15 V, respectively. In addition, as in Figure 2b, the current–voltage characteristics of the ZnO TFTs were retraced using the power-law-term-corrected relation *I_d_*^1/(*α*+1)^ = (*μ*_0_ *C_i_ WL*^−1^*V_ds_*)^1/(*α*+1)^ (*V_gs_* − *V_th_*), and the threshold voltage was re-extracted. Surprisingly, the power-law-term-corrected transfer curve changed to a relatively linear curve, enabling us to extract the threshold voltage with the gate-bias-independent value of 15 V. In addition, the value was consistent with the value from the modified extraction plot of *I_ds_/g_m_*. Thus, we regard the extracted threshold voltages as reliable. However, it is still unconfirmed whether the threshold voltage is genuine. As aforementioned, the conduction states in disordered semiconductor-based TFTs are generally difficult to determine. Hence, to validate that the extracted threshold voltage is authentic, we investigated and carefully analyzed the temperature-dependent current–voltage characteristics of the ZnO TFTs from 180 to 300 K.

**Figure 3:**
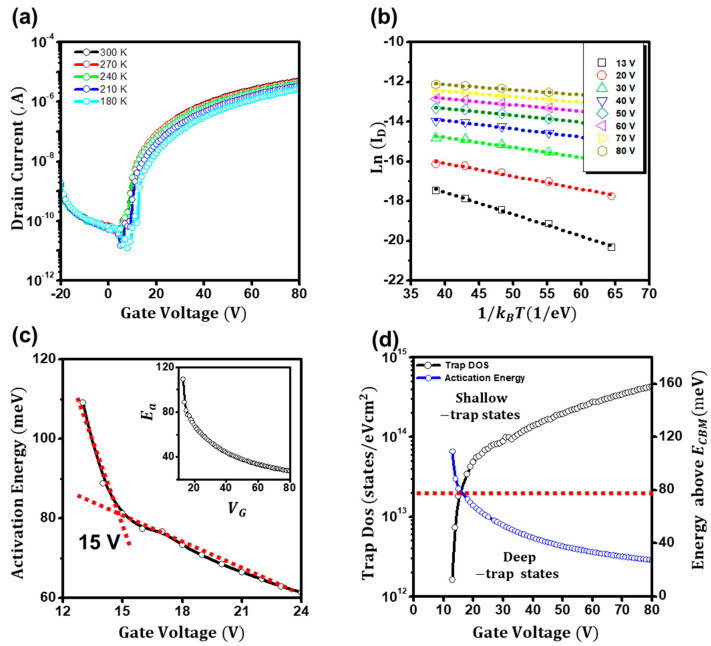
(**a**) Temperature dependence of the transfer curves of the ZnO TFTs. (**b**) Plots of Ln(*I_D_*) as a function of 1/T of the ZnO TFTs. (**c**) Extracted activation energies as a function of gate voltage. Inset: Extracted activation energies for the gate voltage up to 80 V. (**d**) Extracted charge trap density of states (DOS) of the ZnO TFTs.

Figure 3a depicts the temperature-dependent transfer characteristics of the ZnO TFTs. Fundamentally, charge carriers in transistors (including MOSFETs and TFTs) are thought to be mainly transported by the diffusion of carriers below the threshold voltage and by the drift of carriers above the threshold voltage [11,17,31]. If one can decouple the diffusion current from the total drain current, the threshold voltage of the TFTs can be determined [32]. However, direct decoupling in disordered semiconductor-based TFTs is hardly conducted because both diffusion and drift currents occur through thermally activated transports. To this end, we conceived that if the activation energies for conduction were extracted and compared, the threshold voltage of the TFTs could be determined. The required activation energies for diffusion and drift are different. Hence, using the Meyer–Neldel rule of *I(V_gs_) = I*_0_ *exp*(−*E_a_/k_BT_*), we investigated the activation energies (*E_a_*) as in Figure 3b [28,29]. Figure 3c displays extracted activation energies (*E_a_*) as a function of the gate voltage. Notably, at the low-gate-voltage regime (*V_gs_* < 15 V), the activation energy (*Ea*) was deduced to be as high as 110 meV, but it sharply decreased at the gate voltage of 15 V and gradually decreased to ~40 meV (*V_gs_* > 40 V). Although a detailed study on activation energies for diffusion and drift currents in ZnO films is not provided in this study, this abrupt decrease in activation energy at the threshold voltage is attributed to the conduction route change from diffusion to drift. The diffusion current, which is the charge transport driven by the concentration gradient, requires a higher activation energy than the drift current, which is driven by electrical potential. In addition, the deduced voltage of 15 V was consistent with the value from the modified extraction plot. Furthermore, the conduction route change was reconfirmed by investigating the localized (trap) density of states (DOS), *g(E)*, of the ZnO TFTs. Assuming the gate-dependent activation energy is closely related to the energetic difference between the Fermi level and conductive states, the areal density of states of the ZnO TFTs is extracted using following equation [29,30]:

(5) $$g\left(E\right)=\frac{C_{i}}{q}{\left(\frac{dE_{a}}{dV_{gs}}\right)}^{-1}$$

where *E_a_* is the gate-dependent activation energy from the Meyer–Neldel rule *I(V_gs_) = I*_0_ *exp(−E_a_/k_BT_)*. As illustrated in Figure 3d, at the low-gate-bias regime (*V_gs_* < 15 V), as the gate bias increased, the trap density was estimated to be as low as ~10^12^ states eV^−1^cm^2^, but it increased rapidly to ~10^13^ states eV^−1^cm^2^. However, at the high-gate-bias regime (*V_gs_* > 15 V), it was estimated to be as high as ~10^14^ states eV^−1^cm^2^, but it slowly increased. Although trap states are literally trap states, the trap states in ZnO films can act as conduction states in the forbidden gap. Thus, the conduction occurs as follows: below the threshold voltage, the density of the mobile charges that involve the conduction rapidly increases, causing the conduction by vertical state-to-state hopping. In contrast, above the threshold voltage, the density of the mobile charges is saturated; thus, horizontal state-to-state hopping leads to the conduction. This vertical to horizontal hopping change occurs due to the conduction route change, from concentration-limited current (diffusion) to electric-field-driven current (drift). In addition, the deduced voltage of the ZnO TFTs from the low-temperature analysis, 15 V, was consistent with the voltage from the modified extraction plot of *I_ds_/g_m_*. Thus, we can conclude that the threshold voltage acquired by the proposed method is reliable and genuine.

**Figure 4:**
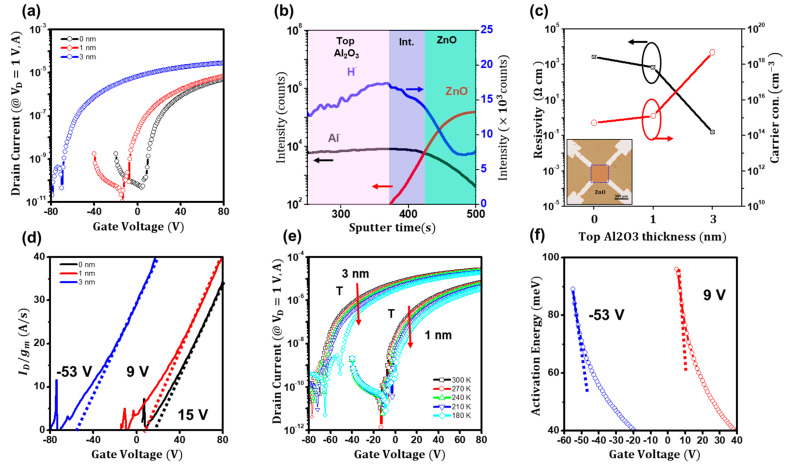
(**a**) The transfer curves of the Al_2_O_3_-coated ZnO TFTs. (**b**) The SIMS depth profiling of the Al_2_O_3_-coated ZnO film. (**c**) Conductivity and carrier concentrations of the ZnO films with the top Al_2_O_3_ layer. (**d**) The modified *I_ds_/gm* plot for the A_l2_O_3_-coated ZnO TFTs. (**e**) Temperature dependence of the transfer curves of the Al_2_O_3_-coated ZnO TFTs. (**f**) Extracted activation energies as a function of gate voltage for the Al_2_O_3_-coated ZnO TFTs.

To further ensure that the threshold-voltage extraction method is applicable for threshold-voltage-shifted ZnO TFTs, we fabricated Al_2_O_3_-coated ZnO TFTs. Figure 4a shows the transfer characteristics (*I_ds_* vs. *V_gs_* at *V_ds_ =* 1 V) of the Al_2_O_3_-coated ZnO TFTs, clearly displaying the threshold voltage shifts. As reported elsewhere, the top Al_2_O_3_ layer on the ZnO films causes conductive changes in the films and leads to significant threshold voltage shifts in the TFTs [23]. Aluminum and hydrogen can offer additional electrons in ZnO films [33]. The cyclic atomic deposition process for the top Al_2_O_3_ layer is conducted at 110 °C for hours; hence, the ions can diffuse into the ZnO side, leading to electron doping. The time-of-flight secondary ion mass (SIMS) analysis in Figure 4b shows that aluminum (Al-) and hydrogen (H-) ions significantly diffused into the films. In addition, Hall measurements of the films in Figure 4c display that conductive ZnO films were formed by the Al_2_O_3_ deposition. As the thickness of the top Al_2_O_3_ layer increased, the carrier concentration rose from ~10^15^ to ~10^19^ cm^−3^ and the resistivity decreased from ~10^4^ to ~10^−1^ ohm-cm. As seen in Figure 4d, using the modified extraction method of *I_ds_/_gm_*, the threshold voltages for 1 and 3 nm thick Al_2_O_3_-coated ZnO TFTs were successfully determined by 9 and −53 V, respectively. Moreover, when investigating the temperature-dependent transfer characteristics of the Al_2_O_3_-coated ZnO TFTs in Figure 4e, the activation energies (*E_a_*) of the 1 and 3 nm thick Al_2_O_3_-coated ZnO TFTs in Figure 4f showed abrupt decreases at 9 and −53 V, respectively. In addition, the power law exponent revealed the degree of the localization of the conduction states. The exponent of pure ZnO TFTs was estimated to be 0.92, whereas they were 0.87 and 0.85 for the 1 and 3 nm thick A_2_O_3_-coated ZnO TFTs, respectively. Notably, the field-effect mobility of pure ZnO TFTs was 3.1 cm^2^V^−1^s^−1^, but it increased to 3.9 and 7.1 cm^2^V^−1^s^−1^ for the 1 and 3 nm thick Al_2_O_3_-coated ZnO TFTs, respectively. This increase in mobility as the exponent decreases is attributed to the conduction states of the charge transport. When the conduction takes place in deep localized states, the charge transport is strongly dependent on the gate bias, resulting in a high power-law exponent. However, when in shallow localized states (due to doping), the charge transport is less affected by the gate bias, leading to a low power-law exponent. The device parameters are listed in Table 1.

Moreover, to verify that the method is applicable for amorphous semiconductors, TFTs based on a well-known amorphous semiconductor, indium–gallium–zinc oxide (IGZO), were fabricated and their threshold voltage was investigated. The bottom-gate top-contact sputter-deposited IGZO TFTs on a chemically cleaned 200 nm thick p^+^-Si/SiO_2_ wafer displayed typical n-type transfer characteristics (*I_ds_* vs. *V_gs_* at *V_ds_* = 1 V), as seen in Figure S2a; the extrinsic field-effect mobility from the transconductance increased, reaching 15.2 cm^2^V^−1^s^−1^ at *V_gs_* = 40 V. As seen in Figure S2b, the threshold voltage (*V_th_*) of the IGZO TFTs also varied with the gate bias, which was estimated to be 17, 22, and 27 V for the gate biases of 25, 30, and 40 V, respectively. However, these arbitrary threshold voltages changed to the definitive value of 14 V after applying the modified extraction method, *I_ds_/g_m_*, as in Figure S2c. In addition, the power-law-term-corrected current relation of the IGZO TFTs also showed a distinctive linear curve in Figure S2d, and the threshold voltage was again deduced to be 14 V. It was also consistent with the value from the modified extraction plot of *I_ds_/g_m_*. As a result, we can state that the threshold voltages of disordered oxide transistors can be easily determined from the transfer curve at room temperature using the proposed threshold-voltage extraction method of *I_ds_/g_m_*.

## 4. Discussion

**Figure 5:**
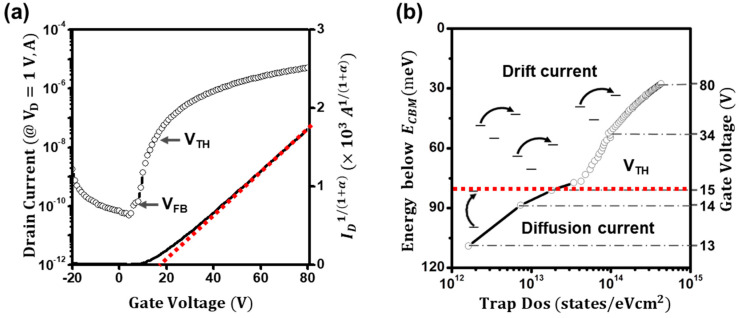
(**a**) The transfer curves and the power-law-term-removed threshold-voltage extraction of the ZnO TFTs. (**b**) Extracted charge trap density of states (DOS) of the ZnO TFTs.

Based on the experiments and findings, we can now understand the operation of the accumulation-mode ZnO TFTs, in which threshold and flatband voltages can be determined due to the deep and shallow trap states. As seen in Figure 5a,b, below the flatband voltage, which is estimated with the turn-on voltage [32], charges cannot be transported, because the induced charges are mainly assigned to fill the deep trap states. However, as the gate bias increases above the flatband voltage, deep trap states are filled with gate-induced charges, and the charges can be transported. However, the charges are transported through the deep trap states, mainly transported by concentration-gradient-driven currents (diffusion current). Diffusion currents occur due to the inverted charges in the depletion region of MOSFETs and due to accumulated charges in the deep trap states of TFTs. When the gate voltage further increases above the threshold voltage, the deep trap states are almost filled with the charges, enabling electric-field-driven currents to transport the charges through the shallow trap states (drift current). Capacitance–voltage (C–V) measurements could be used to characterize the interfacial defects of TFTs [14]. However, in this case, the deposition of aluminum electrodes on ultra-thin atomically deposited ZnO films for a metal–oxide–semiconductor structure causes electron doping [33] and alters the threshold voltage of the disordered TFTs. For this reason, we attempted to detect the trap states of the ZnO TFTs using the TFTs’ structure, by measuring their low-temperature characteristics. Additionally, proving that the method is applicable more generally would require threshold-voltage extraction using other semiconductors, such as p-type or ambipolar semiconductors. Nevertheless, based on the results, we believe that the proposed method is reliable and applicable to determine the threshold voltages of disordered oxide semiconductor TFTs.

## 5. Conclusions

In this study, the threshold-voltage extraction methods of bottom-gate atomic-deposited ZnO TFTs were investigated. The ZnO TFTs display typical n-type enhancement-mode transfer characteristics, but the threshold voltage is difficult to determine. By considering that the obscure threshold voltage is attributed to the localized states, we derived current–voltage relations decoupled with the gate-bias-dependent factor and successfully extracted the reliable threshold voltage. Furthermore, to ensure the threshold voltage was genuine, the temperature-dependent current–voltage characteristics of the ZnO TFTs were investigated. Using the Meyer–Neldel rule, the activation energy as a function of the gate voltage was deduced. Notably, the activation energy significantly changed at the threshold voltage, which is attributed to the conduction route change from diffusion to drift. The diffusion current below the threshold voltage required higher energy than the drift current. More dramatically, the change in density of the ZnO TFTs states also revealed the conduction route change. The trap density increased rapidly from 10^12^ to 10^13^ states eV^−1^cm^2^ below the threshold voltage, whereas it saturated to 10^14^ states eV^−1^cm^2^ above the threshold voltage. This significant change in trap density implies that the increment in the mobile charges released from the deep trap state led to conduction at the low-voltage regime (diffusion), while the electric-field-affected mobile charges resulted in conduction at the high-gate-bias regime (drift). We thus conclude that our proposed method offers practical and theoretical benefits for extracting the threshold voltage and understanding the operation of disordered accumulation-mode ZnO TFTs.

## Figures and Tables

**Table 1:** Summary of the device parameters for the ZnO and Al_2_O_3_-coated ZnO TFTs.

	*μ* (cm^2^V^−1^s^−1^)	Power-Law Exponent	*V_turn-on_* (V)	*V_th_* (V)		
				Linear Fitting (*V_gs_*)	*I_ds_/g_m_*	*E_a_* Analysis
ZnO	3.1	0.92	8	25/36/42 (40/60/80)	15	15
1 nm thin Al_2_O_3_-coated ZnO	3.9	0.87	−5	21/28/41 (40/60/80)	9	9
3 nm thin Al_2_O_3_-coated ZnO	7.1	0.85	−72	−42/−26/−18 (−20/20/80)	−53	−53

## Data Availability

Not applicable.

## Supplementary Materials

The following supporting information can be downloaded at: https://www.mdpi.com/article/10.3390/ma16082940/s1, Figure S1: Device parameter analyses of ZnO TFTs with a four-point probe configuration; Figure S2: Device parameter analyses of IGZO TFTs.
