# Supplementary material for: Threshold-Voltage Extraction Methods for Atomically Deposited Disordered ZnO Thin-Film Transistors
Source: Materials (Basel). 2023 Apr 7;16(8):2940. doi: 10.3390/ma16082940 (PMC10144086; doi:10.3390/ma16082940)
Supplement: Supplementary file 1 [file materials-16-02940-s001.zip › materials-2294770-supplementary.pdf]

materials

MDPI

Supplementary Materials

Threshold-Voltage Extraction Methods for Atomically Deposited Disordered ZnO Thin-Film Transistors

Minho Yoon

Department of Physics and Institute of Quantum Convergence Technology, Kangwon National University, Chuncheon 24341, Republic of Korea; minhoyoon78@gmail.com

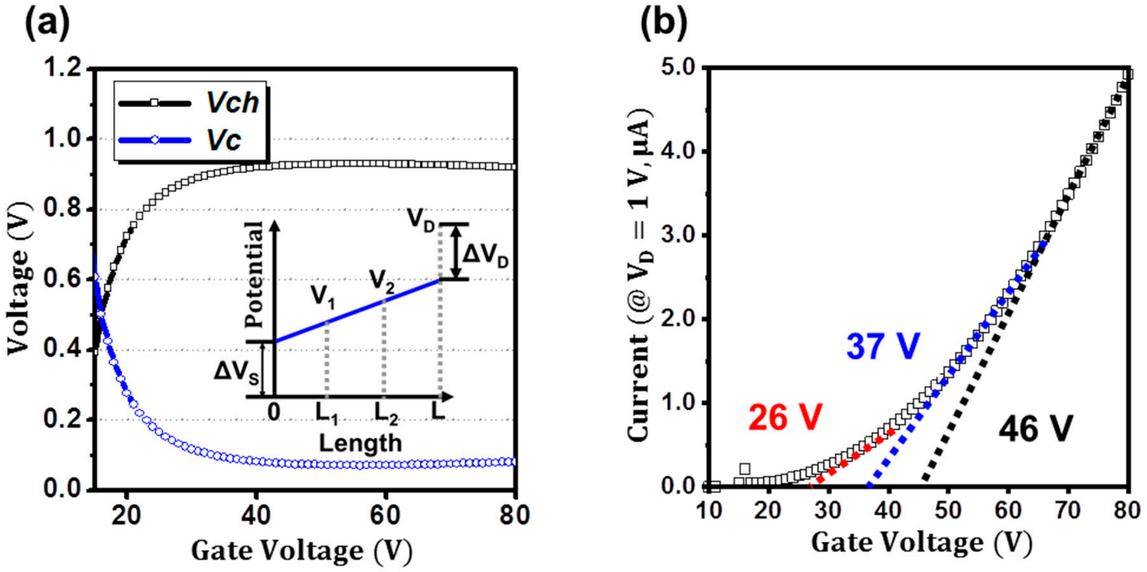

Figure S1. (a) Potential distribution of the ZnO TFTs with a four-point probe configuration. Inset: Schematic illustration of the four-point probe method. (b) The threshold voltage extraction using the intrinsic current–voltage characteristics with the gate bias range of 40 (red), 60 (blue), and 80 V (black).

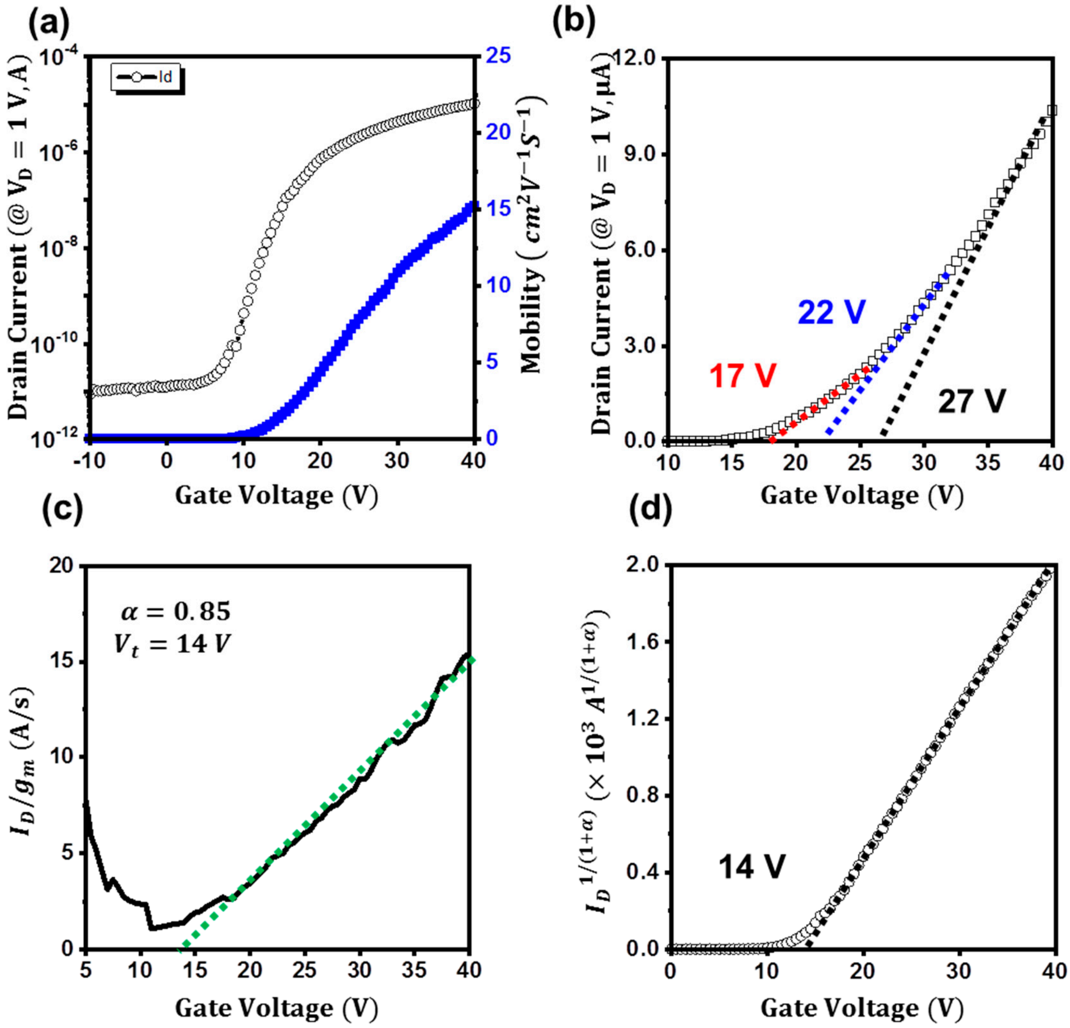

Figure S2. (a) Transfer characteristics (I_{ds} vs. V_{gs}) of the IGZO TFTs. (b) The threshold voltage extraction of the IGZO FET with the gate bias range of 25 (red), 30 (blue), and 40 V (black). (c) The modified plot of *Ids*/*gm* for the IGZO TFTs. (d) The modified threshold voltage extraction of the IGZO TFTs.
